# Strangulated or incarcerated spontaneous lumbar hernia as exceptional cause of intestinal obstruction: case report and review of the literature

**DOI:** 10.1186/1749-7922-9-44

**Published:** 2014-07-16

**Authors:** Marcus Fokou, Patrick Fotso, Marcelin Ngowe Ngowe, Arthur Essomba, Maurice Sosso

**Affiliations:** 1 Departement of Surgery, Yaounde General Hospital, POB 5408, Yaounde, Cameroon

**Keywords:** Lumbar hernia, Strangulation, Incarceration, Review

## Abstract

Lumbar hernias are rare conditions and about 300 cases have been reported since the first description by Barbette in 1672. Therefore strangulation or incarceration are also exceptionally encountered. We present a 62 -year-old-man who had strangulated left lumbar hernia and consequent mechanical small-bowel obstruction, alongside with a non strangulated right lumbar hernia. Through a median laparotomy, an intestinal necrosis was found. A bowel resection with end to end anastomosis was performed and the lumbar hernias were repaired on both sides. The recovery was uneventfull. To the best of our knowlwdge thanks to the litterature review presented here, this is the 19th case of incarcerated or strangulated spontaneous lumbar hernia described in the surgical litterature since 1889.

## Introduction

Lumbar hernia though well described, is a rare condition with approximately 300 cases reported in the literature since it was first described by Barbette in 1672. Twenty percent of lumbar hernias are congenital and the other 80% are acquired; the acquired lumbar hernias can be further classified into either primary (spontaneous) or secondary (either iatrogenic or traumatic) [1]. It may occur bilaterally or in association with another hernia, mostly inguinal hernia. Due to its rarity, complications such as bowel obstruction secondary to incarceration or strangulation are also exceptionally reported and therefore there is no specific management guideline [2]. The case presented here was in association with a controlateral non strangulated lumbar hernia. To the best of our knowlege this is the 19th case of strangulated or incarcerated spontaneous lumbar hernia reported in the surgical litterature since the case published in the BMJ by Hume in July 1889 [3].

## Case report

**Figure 1:**
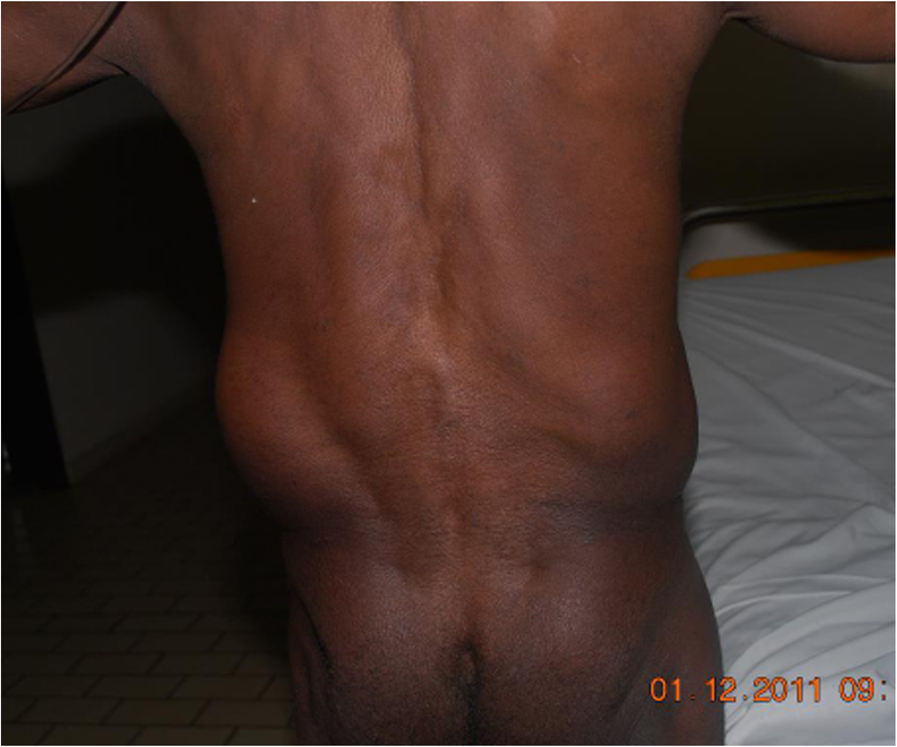
Clinical aspect of the pateient with bilateral lumbar swelling.

**Figure 2:**
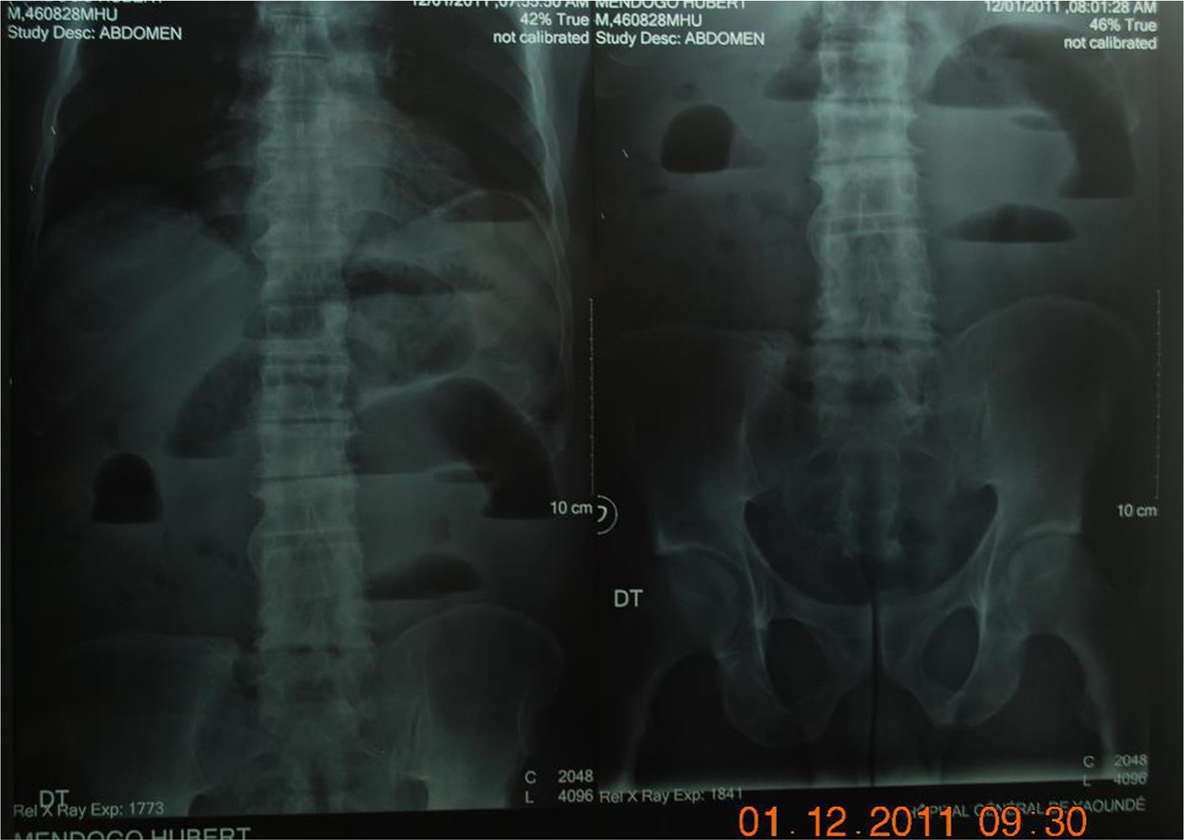
Plain upright abdominal X-ray, taken preoperatively demonstrates Gas shadow in the anabdomen.

A 62-year-old man presented to our emergency department with nausea, vomiting and abdominal pain together with swelling and pain of the left lumbar region for 4 days. His medical history was not consistent he was a farmer. On physical examination, the abdomen was distended and tympanic. There was tenderness, especially in the left lumbar regiont. A small painfull irreductible mass (about 6-cm in diameter) was palpated above the left iliac crest. Another mass, instead reductible was found on the right lumbar region above the iliac crest (Figure 1). Abdominal roentgenograms in the upright position revealed multiple dilated loops of small intestine with air–fluid levels (Figure 2). An ultrasound of the mass revealed the presence of non parietal tissue and the communication with the abdominal cavity.

A preoperative work-up was normal except the ESR CRP and leukocyte count that were increased. Electrolyte and other biochemical studies were within normal limits.

The patient was taken to the operating room for urgent surgery with the diagnosis of intestinal obstruction due to incarcerated lumbar hernia. An abdominal exploration was performed through a midline incision. During the exploration, at approximately 200 cm from the Treitz ligament, a loop of small bowel was found incarcerated within the left lumbar triangle of Petit. A 40-cm necrotic small-intestinal loop was resected and continuity was re-established. During evaluation of the hernial areas, there was no other herniation except the right lumbar hernia already mentioned. The lumbar hernias were repaired with a 2(USP) resorbable suture.

The post-operative period was uneventfull. The patient was discharged without any complication on the thirteen postoperative day. As of date more than 2 years after the operation, the patient is doing well. No recurrence has been observed.

## Discussion

Lumbar hernia is a well documented but extremely rare condition. Men in their sixth decades and above are more proned than women. Complications such as strangulation is rarely encountered and since 1889 with the excellent description of a patient having a strangulation by Hume; surgeon at the Royal Infirmary in Newcastel on Tyne [3], about 17 other cases have been reported till date [4-14] making our case the 19th (Table 1).

**Table 1:** Cases of strangulated or incarcerated spontaneous lumbar hernia reported since 1889

**Year**	**Patient (age, sex)**	**Type of lumbar hernia**	**Organ involved**	**Author (reference )**
1889	68 male	Petit	Small intestine	Hume (3)
1955	-	-	-	Makhoovos (4)
1959	71 male	Petit	Richter hernia	Glyn Millard (5)
1971 (2 cases)	-	-	-	Florer (6)
1974	-	-	-	Emakov (7)
1986	-	Petit	Colon	Horovitz (8)
1987	-	-	-	Carrelet (9)
1989 (6 cases)	Mean age 67 males	Pettit 5 Gynfelt 1	Small intestine	Mbakor (10)
1989	70, female	Petit	Colon	Hide IG (11)
2003	70 female	Petit	Colon	Astarcioğlu (12)
2010			Colon	Light (13)
2010	79 female	Petit	Small bowel	Teo (14)
2014	62 male	Petit	Small bowel	Current case

Lumbar hernia is seen mostly in association with other abdominal wall hernias in elderly patients . They can also be bilateral as seen in this case. It was reported that coexistence of lumbar hernia and other abdominaal wall Hernia is observed in 13% of patients. These reports suggest that a patient presenting with a lumbar hernia should be explored for the presence of a coexisting hernia, such as inguinal, femoral or obturator hernia [1]. In our case, except the controlateral lumbar hernia, no other type of abdominal wall hernia was seen.

Preoperative diagnosis of lumbar hernia is common. Because specific physical findings are obvious, They are usually confused with lipoma or other superficial swelling of the flank. Unfortunately the diagnosis can be delayed and done after bowel obstruction. This was the case in our patient who was presenting signs of bowell obstruction before the lumbar hernia was identified. In some cases it is during diagnostic laparotomy for bowel obstruction that the diagnosis is done as also for abdominal wall hernias [1,2].

Modern radiological modalities such as CT Scan, ultrasonography (US) and magnetic resonance imaging (MRI) can reliably make the early diagnosis of lumbar hernia, especially in elderly and frail patients having other abdominal wall hernias [1]. X-ray films may be usefull only in case of bowel obstruction as in our case, But CT and US can be applied to intestinal obstructions in which the origin is obscure [11-13].

Modern hernia repair using synthetic graft is recommended in lumbar hernia. But in case of strangulation, an incision for exploration or diagnostic laparoscopy should be preferred. In this patient, we perfomed a laparotomy since the patient presented late. Actually there are enough evidence that in abdominal wall hernias mortality is most often associated with delay in presentation and diagnosis [2]. This can probably apply to lumbar hernia even though there is no specific study addressing that specific issue.

Intestinal obstruction and bowel necrosis, require emergency laparotomy with a midline incision. This approach gives the best exposure, allows reduction of the hernial content and facilitates bowel resection and abdominal toilet, if necessary. Other herniation sites can also be evaluated with this incision. Other types of approaches, such as preperitoneal, lumbar, can be applied when early diagnosis is made or in not strangulated cases [1].

A laparoscopic approach was also envisaged. It is currently encouraged in emergency repair of complicated abdominal wall hernias [2]. However, this approach may prolong the time of operation and increase the risk of mortality in centers that have limited laparoscopic experience and in patients having a bad general condition.

Various repairs include primary suture of the orifice, muscle flaps, omentum, broad ligament, uterine fundus, prosthetic material and mesh plug. Without repair, compications rates of approximately 25% are reported [1].

The use of mesh for repair of the strangulated hernias in which resection was performed is controversial [2]. Some authors do not recommend this type of repair due to the higher risk of rejection caused by infection. Others recommend it when an intestinal resection is carried out with sufficient care to minimize infective complications; therefore, the use of mesh will not be contraindicated [2,4,9]. In our practice we don’t use prosthetic material in strangulated hernias and particularly like in this case where a bowell resection was performed.

Mortality is reported to be between 10% and 50% in lumbar hernia. Unfavorable outcomes are commonly associated with delay in diagnosis and therapy, poor condition, elderly patients having coexistent diseases and strangulation with intestinal gangrene [1,14].

Although lumbar hernias are rare, they should be considered when an elderly, thin patient presents with a bowel obstruction. Early diagnosis and treatment are the most important factors in decreasing mortality and morbidity; therefore, rapid action for diagnosis and therapy is essential.

## Consent

Written informed consent was obtained from the patient for the publication of this report and any accompanying images.

## Competing interests

The authors declare that they have no competing interests.

## Authors’ contributions

MF, Conceived and wrote the manuscript. PF, Collected the data. AE, MNN, MS critically revised the manuscript. Overall responsibility MF. All authors read and approved the final manuscript.
